# The global landscape of clinical trials and drug discovery for brain metastasis

**DOI:** 10.1186/s12967-024-05310-8

**Published:** 2024-08-06

**Authors:** Jiatong Ding, Yale Jiang, Jiawei Zhou, Qiyu Tang, Shujun Xing, Shuhang Wang, Ning Li

**Affiliations:** Clinical Trials Center, National Clinical Research Center for Cancer/National Clinical Research Center for Cancer/Cancer Hospital, Chinese Academy of Medical Sciences and Peking Union Medical College, Beijing, 100021 China

To the editor,

Brain metastasis (BrM) remains a serious complication of systemic cancer due to its consistent association with poor clinical outcomes [1]. Given the increasing incidence but unsatisfactory clinical outcomes of BrM, many new therapies have been tested in clinical trials to explore reliable strategies [2]. Here, we present a comprehensive analysis of the interventional clinical trials including patients with BrM during the last 10 years. The classification and targets of drug interventions for BrM treatment in these trials were also analyzed to provide supporting data for future research on treatment of BrM.

Using ClinicalTrials.gov database, a total of 434 eligible interventional BrM clinical trials were identified from Jan 1, 2013, to Dec 1, 2023, based on the inclusion criteria (Methods in the Supplement). The annual number of trials maintained at a relatively stable level in recent years, which reached its highest peak in 2021 but showed a downward trend in the past two years (Fig. 1A). Phase II trials accounted for the highest proportion (202, 46.5%), while the proportions of phase III and IV trials are only 8.3% (36) and 0.7% [3]. Most trials (194, 44.7%) included multiple primary tumor sites. Among trials focusing on a single primary tumor site, non-small cell lung cancer (NSCLC) (99, 22.8%), breast cancer (77, 17.7%), and melanoma (46, 10.6%) were the most frequent cancer types (Fig. 1B). NSCLC also occupied the highest proportion (15, 41.7%) in phase III trials. There were only three phase IV trials that covered BrM from NSCLC, breast cancer and unspecific primary tumor sites (Fig. 1C).

For drug treatment mode, 286 clinical trials involving pharmaceutical interventions were included. Among them, target therapy (192, 67.1%), immunotherapy (90, 31.5%), and chemotherapy (76, 26.6%) have attracted more attention. Moreover, target therapy and immunotherapy showed a trend of occupying higher proportions (Fig. 1D). The peptide-drug conjugates (PDC) and nanoparticles (NP) have also gained certain attention. In breast cancer BrM, endocrine therapy appears to become an available combination partner of target therapy. Target therapy also constituted the most widely used treatment modality in BrM from NSCLC, breast cancer, and unspecified solid cancer, but immunotherapy accounted for the highest proportion in melanoma (Fig. 1E). Apart from the classic targets, such as PD-1 (programmed death-1) and EGFR (epidermal growth factor receptor), novel targets were also tested in recent trials (Table 1). For cell therapies, dendritic cells, NK cells, tumor-infiltrating lymphocytes, autologous progenitor expansion T cells, CAR-T cells, personalized cellular vaccine, peripheral blood mononuclear cells, and TCR-gene engineered lymphocytes are emerging.

Notably, most of the clinical trials have tested combination regimens including drug treatment and radiotherapy. More confirmatory evidence is needed to determine the optimal sequence of treatment options. Drug delivery systems have also attracted much attention in recent years. For example, ANG1005, the most well-known brain permeable PDC, has entered a phase III clinical trial (NCT03613181) for BrM and leptomeningeal disease [2]. PDC has its advantages in tumor penetration, production cost and immunogenicity, making it a research hot spot and a promising track for investment [4]. The theragnostic potential of NPs has also been highlighted in the management of BrM, while researches on the clinical applicability of NPs for BrM are still in early stages (for example, NCT04899908 and NCT05255666).

In conclusion, the clinical trials for BrM have developed rapidly worldwide, but still at early exploratory stages. Due to the biological heterogeneity, the potential differences in BrM between cancer types should receive more attention. With research on novel therapeutic strategies against BrM gathering momentum, several new drugs targeting different molecules and drug delivery approaches have entered clinical trials, which is expected to increase the treatment opportunities for BrM patients.

**Fig. 1:**
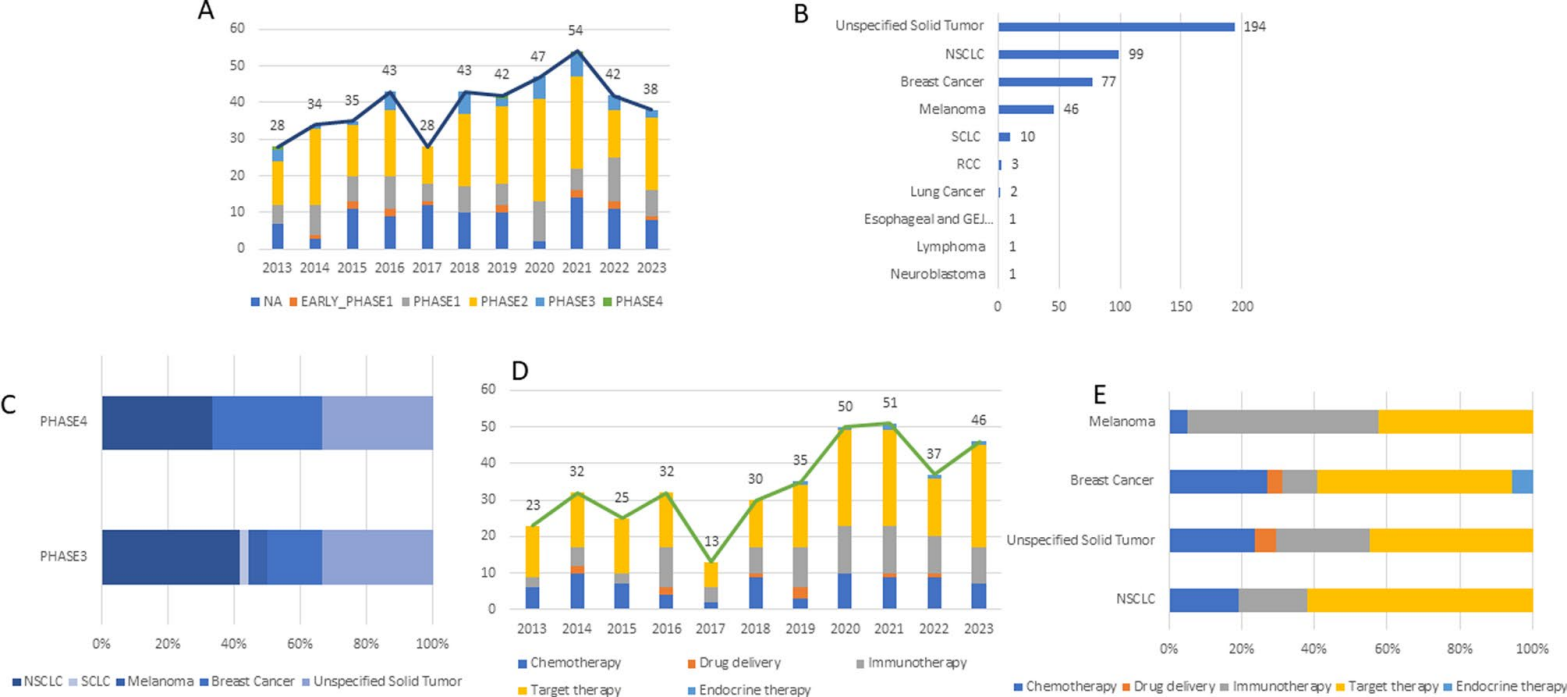
The landscape of clinical trials and drug discovery for brain metastasis. (**A**) Annual numbers of the clinical trials registered worldwide in ClinicalTrials.gov. (**B**) Interventional clinical trials for brain metastasis across primary tumor types. (**C**) Phase III and phase IV clinical trials across primary tumor types. (**D**) Annual numbers of the clinical trials across different drug therapy strategies. (**E**) Drug treatment across primary tumor types

**Table 1:** Recent clinical trials involving drugs targeting novel therapeutic targets

NCT Number	Primary tumor site	Phases	First Posted	Drug interventions	Target
NCT05620914	Unspecified Solid Tumor	EARLY_PHASE1	2022	Patritumab deruxtecan	HER3
NCT05704933	Melanoma	EARLY_PHASE1	2023	Nivolumab, Ipilimumab, Relatlimab	PD-1 + CTLA-4 + LAG3
NCT03796273	Breast Cancer	EARLY_PHASE1	2019	Ketoconazole	tGLI1
NCT02429570	Unspecified Solid Tumor	NA	2015	Meclofenamate	FTO
NCT03423628	Unspecified Solid Tumor	PHASE1	2018	AZD1390	ATM
NCT02589522	Unspecified Solid Tumor	PHASE1	2015	Berzosertib	ATR
NCT06137651	Breast Cancer	PHASE1	2023	Trotabresib, Vinorelbine	BET
NCT02215512	Unspecified Solid Tumor	PHASE1	2014	RRx-001	CD47 + SIRP-α
NCT04396717	Unspecified Solid Tumor	PHASE1	2020	Pritumumab	EDV
NCT05669352	Melanoma	PHASE1	2022	CA-4948, Pembrolizumab	IRAK-4 + PD-1
NCT04430842	Unspecified Solid Tumor	PHASE1	2020	QBS10072S	LAT1
NCT04250545	NSCLC	PHASE1	2020	Sapanisertib, Telaglenastat Hydrochloride	mTOR + GLS1
NCT04789668	Unspecified Solid Tumor	PHASE1	2021	Bintrafusp Alfa, Pimasertib	PD-L1/TGF-β + MEK
NCT04631029	SCLC	PHASE1	2020	Atezolizumab, Carboplatin, Entinostat, Etoposide	PD-L1 + HDAC
NCT05789589	Unspecified Solid Tumor	PHASE1	2023	Azeliragon, Corticosteroid	RAGE
NCT06128148	Unspecified Solid Tumor	PHASE1	2023	JYP0322	ROS1
NCT04334863	Unspecified Solid Tumor	PHASE1	2020	WP1066	STAT3
NCT04460937	Esophageal and GEJ Cancer	PHASE1	2020	Adavosertib	Wee1
NCT05866432	Breast Cancer	PHASE2	2023	Datopotamab deruxtecan	TROP2
NCT02014545	NSCLC	PHASE2	2013	Lucanthone	APE-1
NCT03964090	Secondary Central Nervous System Lymphoma	PHASE2	2019	TEDD-R, TEDDI-R, Ibrutinib, Cytarabine, Isavuconazole, Methotrexate	BTK + CYP3A4 + CD20
NCT04899921	Melanoma	PHASE2	2021	Ipilimumab, Nivolumab, Troriluzole	EAAT2 + PD-1 + CTLA-4
NCT05999357	NSCLC	PHASE2	2023	JDQ443	KRAS G12C
NCT04460729	NSCLC	PHASE2	2020	Capmatinib	MET
NCT02595905	Breast Cancer	PHASE2	2015	Cisplatin, Veliparib	PARP
NCT02452294	Melanoma	PHASE2	2015	Buparlisib	PI3K
NCT05909618	Melanoma	PHASE2	2023	Crizanlizumab-Tmca, Nivolumab	P-selectin + PD-1
NCT05746481	NSCLC	PHASE2	2023	Tiragolumab, Atezolizumab, Pemetrexed, Carboplatin	TIGIT + PD-L1
NCT04647916	Breast Cancer	PHASE2	2020	Sacituzumab Govitecan	Trop-2
NCT04674683	Melanoma	PHASE3	2020	HBI-8000, nivolumab	HDAC + PD-1

GEJ, gastroesophageal junction; NSCLC, non-small cell lung cancer; SCLC, small cell lung cancer

## Electronic supplementary material

Below is the link to the electronic supplementary material.

Supplementary Material 1

## Data Availability

All data used and/or analyzed in this manuscript is publicly available on the ClinicalTrials.gov database (https://clinicaltrial.gov/).
