# Supplementary material for: The global landscape of clinical trials and drug discovery for brain metastasis
Source: J Transl Med. 2024 Aug 6;22:744. doi: 10.1186/s12967-024-05310-8 (PMC11304579; doi:10.1186/s12967-024-05310-8)
Supplement: Supplementary file 1 — Supplementary Material 1 [file 12967_2024_5310_MOESM1_ESM.docx]

Method:

The clinical trial studies were initially downloaded from the ClinicalTrials.gov database using the following search terms: (Brain metastasis) AND (First posted date is form January 1, 2013, to December 1, 2023) AND (Study type is interventional). The studies were removed if they investigated on non-BrM diseases or if they did not include treatment arm descriptions. The clinical trials with ambiguous phase descriptions were corrected using the following logic: trials with both phase one and phase two status were treated as trials of phase one, and trials with both phase two and phase three status were treated as trials of phase two. Finally, the targets of pharmaceutical interventions were obtained from the PubMed database.
